# Processing of Bathymetric Data: The Fusion of New Reduction Methods for Spatial Big Data

**DOI:** 10.3390/s20216207

**Published:** 2020-10-30

**Authors:** Marta Wlodarczyk-Sielicka, Wioleta Blaszczak-Bak

**Affiliations:** 1 Department of Navigation, Maritime University of Szczecin, Waly Chrobrego 1-2, 70-500 Szczecin, Poland; 2 Faculty of Geoengineering, University of Warmia and Mazury in Olsztyn, Oczapowskiego 1, 10-719 Olsztyn, Poland; wioleta.blaszczak@uwm.edu.pl

**Keywords:** big data applications, bathymetry, data reduction, data processing, data visualization, fusion of spatial data

## Abstract

Floating autonomous vehicles are very often equipped with modern systems that collect information about the situation under the water surface, e.g., the depth or type of bottom and obstructions on the seafloor. One such system is the multibeam echosounder (MBES), which collects very large sets of bathymetric data. The development and analysis of such large sets are laborious and expensive. Reduction of the spatial data obtained from bathymetric and other systems collecting spatial data is currently widely used. In commercial programs used in the development of data from hydrographic systems, methods of interpolation to a specific mesh size are very frequently used. The authors of this article previously proposed original the true bathymetric data reduction method (TBDRed) and Optimum Dataset (OptD) reduction methods, which maintain the actual position and depth for each of the measured points, without their interpolation. The effectiveness of the proposed methods has already been presented in previous articles. This article proposes the fusion of original reduction methods, which is a new and innovative approach to the problem of bathymetric data reduction. The article contains a description of the methods used and the methodology of developing bathymetric data. The proposed fusion of reduction methods allows the generation of numerical models that can be a safe, reliable source of information, and a basis for design. Numerical models can also be used in comparative navigation, during the creation of electronic navigation maps and other hydrographic products.

## 1. Introduction

At present, there are many different sensors that are employed to collect spatial data from water areas. New technological solutions focus on the use of autonomous hydrographic vehicles, which often have different sensors installed, including modern hydroacoustic sensors [1,2,3]. The use of such modern solutions allows the collection of a lot of information, such as that on the location, depth, and distance, at the same time [4,5]. Very often, there is a desire to combine observations obtained from different sources, thus increasing the quality and accuracy of the planned final product [6,7,8]. Due to their mobility and dimensions, floating platforms can operate in shallow areas. These types of autonomics vehicles have become more and more popular [9]. The basic device installed on modern autonomous floating platforms is a multibeam echo sounder (MBES), which sends a hydroacoustic beam towards the bottom and measures the depth under the floating platform [10]. The MBES measures the vertical distance between its transducer and the sea bottom, or an object situated on the sea bottom. The MBES sends several signal beams in multiple directions arranged into a wide swath. The depth value is obtained by calculating the time difference between the moment of transmitting and receiving the hydroacoustic beam after its reflection from the bottom. The sound velocity and the directions of beams are required. It should be mentioned that the bathymetric data obtained from multibeam echosounders are one of the basic data types used in systems modeling of the seafloor [11]. Data obtained from modern hydroacoustic systems are very large sets of bathymetric points belonging to the spatial big data group. The processing and analysis of such large sets are laborious and expensive. MBES data are similar in nature to a point cloud from Light Detection and Ranging (LiDAR) and may be called big data. Multi-beam echosounders generate millions of points during one passage of a hydrographic ship. An echo sounder transducer sends out multiple acoustic beams at different angles, forming a so-called fan of acoustic beams. In this way, a significant band of bottom coverage with measurement data is achieved, guaranteeing almost 100% coverage of the surveyed area. The high resolution of bathymetric data obtained in this way enables a faithful reproduction of the characteristics of the bottom of the basin. The standard methodology of the processing of MBES big data applied to generate digital terrain model (DTM) generally consists of the following stages: (1) obtaining a whole three-dimensional multibeam sonar data set; (2) pre-processing (including, among others, the filtration process, noise removal, and data reduction); (3) DTM construction; and (4) analysis. The reduction of spatial data obtained from bathymetric systems is widely used nowadays.

We can reduce sets of points at the stage of preliminary data preparation (reducing the number of sets of observations by deleting points) or during the generation of numerical models (by interpolation of the depth of points on grid nodes). It is important not to remove characteristic points during the reduction. Reduction methods available in commercial programs, such as random and space methods, are in fact accidental, which means that the algorithm removes every *i*th (*i* = 1, 2,…, *n*) point, without checking its significance [12]. However, in commercial programs used in the processing of hydrographic data, methods of interpolation to a specific mesh size are very frequently used. In this case, the output data do not retain the source characteristics [13]. In this study, the authors focused on processing reduced datasets from real hydroacoustic measurements. This approach can be used in any bathymetric data processing system and is quite different from the standard forms of modeling the seabed surface from high-density data. As mentioned before, known solutions use interpolated values. Our proposed approach retains the actual values, which significantly increases the accuracy and reliability of the reduced data.

## 2. Related Works

The International Hydrographic Organization (IHO) standards should be mentioned here [9]. All bathymetric measurements and the processing of bathymetric data are subject to international IHO regulations. They clearly define the measurement classes and guidelines for the accuracy of the collected and processed data. In the case of the bathymetric grid, international standards define the main gridding methods employed for bathymetric datasets, for example [14,15,16], the shallowest depth, deepest depth, mean, statistical median, weighted mean (calculates an average depth for each grid node; in this method, the inverse to the distance from the sounding location to the nodal position is used as weighting schema), total propagated uncertainty (TPU) weighted mean (uses the elevation and related total propagated uncertainty for each contributing depth estimate to calculate a weighted average depth for each grid node), the combined uncertainty and bathymetric estimator (CUBE) algorithm (uses the elevation and related total propagated uncertainty for each contributing sounding to calculate one or many hypotheses for an area of interest and they are used to estimate statistical representative depths at each grid node), nearest neighbor, natural neighbor interpolation, spline gridding, and kriging.

For navigation safety purposes, the shallowest depth value is often used. The authors assumed, in their research, that the safety of navigation is the most important aspect when creating hydrographic products. When analyzing the current international standards [14,15,16] and the development of data collection systems, it is assumed that, in order to model the shape of the bottom, a set of points with known parameters (XYZ) is required.

The authors proposed original methods of reduction in their previous papers, including the true bathymetric data reduction method (TBDRed) [17] and Optimum Dataset (OptD) method [18], which maintain the actual position and depth for each of the measured points, without their interpolation. The main goal of this article is to propose the fusion of these original reduction methods, which is a new and innovative approach to the problem of bathymetric data reduction. The proposed solution is an innovative approach. Reduction methods proposed by the authors do not interpolate spatial data, but keep their raw characteristics: coordinates and depth. Therefore, one should take into account the fact that the TBDRed and OptD methods leave other points after the reduction, albeit still real ones. It is possible that the points are repeated in the result sets, but duplicates are removed during the merge, so as not to increase the file size and memory space. It can be noted that, thanks to the different points produced after the two reduction methods, the reliability of the integrated dataset increase and the certainty that all points are in integrated dataset are important (from the point of view of the criteria set for each method). The proposed integration is an innovative solution that allows for an accurate representation of the bottom surface, despite data reduction, while maintaining the characteristics of the source data. It should be made clear here that the main thrust of the reduction method is that the input bathymetric data are correctly processed—their position and depth comprise true surveying values. When collecting data using MBES, there are many corrections influencing the accuracy of the measurements which are taken into consideration, such as the water properties, head submersion, errors in the average speed of sound in water, and erroneous offset inputs in the measurement devices. In the next step, which is the initial bathymetric data pressing, there are further errors related to the initial rough filtration and to converting the raw data into a ‘swath’ file. The proposed method of reduction is not related to the processing of data and the accuracy of data collection. Therefore, the accuracy of the input data does not impact the results of reduction.

Thanks to the proposed merger of reduction methods, in addition to the accuracy of the final products, another advantage is the reduction of the data processing time and costs associated with it. Additionally, a reduced data set can be analyzed faster and take up less storage space on the computer. An additional contribution should also be mentioned here, which is the use of artificial neural networks to reduce spatial big data. Commercial programs that are available on the market do not have such a solution. Based on the authors’ previous publications, the use of self-organized artificial neural networks to group bathymetric data is an alternative to classical methods. With the use of optimal parameters, the network learns itself and obtains satisfactory results at the output. The main assumption of the fusion of the proposed reduction method is the preservation of real data XYZ, without interpolation.

## 3. Test Data

This section contains a description of the data sets and the test area that has been selected for fusion-related research on the selected methods. The bathymetric datasets used in the research were gathered by the use of a GeoSwatch Plus 250 kHz multibeam echo sounder and supplementary equipment (GPS/RTK, satellite compass, and motion sensor) on board a Hydrograf XXI laboratory. It should be noted that the measurement profiles were employed to maintain 100% coverage of the measured body of water, in accordance with the special class requirements for hydrographic measurements.

The authors decided to test the proposed solution in two different areas, which differed from each other in terms of the shape of the bottom:

- Test area 1—LNG port,
- Test area 2—Inski canal.

**Figure 1:**
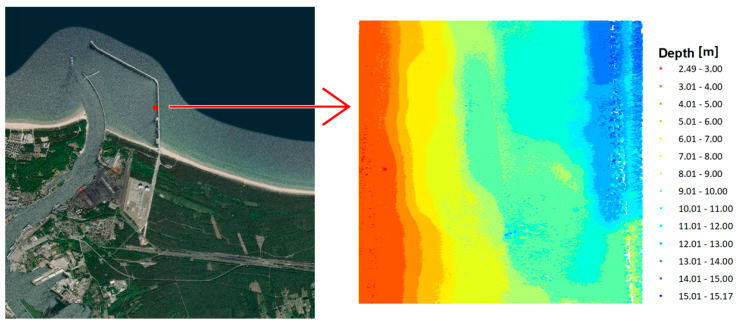
Test area no. 1—LNG port.

Both research areas are located in Poland. The first test area is located in the Baltic Sea and is part of the newly established LNG port in Swinoujscie. The range of the area under study was 100 m × 100 m. The test dataset included a total of 2,670,450 measuring points. The minimum depth in the studied area is 2.49 m, maximum depth is 15.17 m, and mean depth is 7.30 m. The range of spatial data with depth visualization is presented in Figure 1.

**Figure 2:**
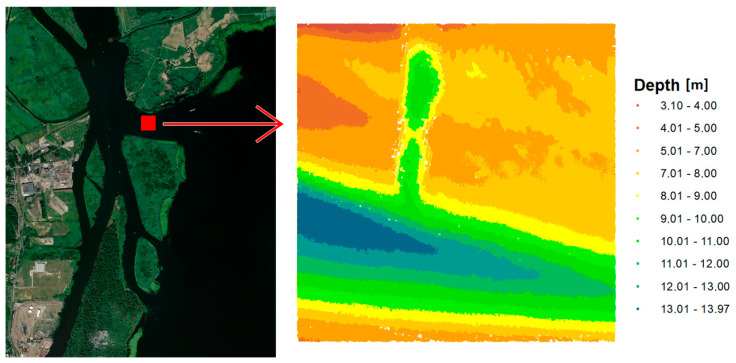
Test area no. 2—Inski canal.

The second test area is located in the area of the port of Szczecin and has different characteristics because it is part of the channel connecting the sea and inland roads. The range of the second test area was larger and was 200 m × 200 m. The test set included a total of 435,805 measuring points. The minimum depth in the tested area is 3.10 m, the maximum depth is 13.97 m, and the average depth is 8.77 m. The range with depth visualization is presented in Figure 2.

The data sets under examination contained many measurement points. The distance between individual points was up to several centimeters. In accordance with international standards, the data were collected and processed in the Universal Transverse Mercator zone 33 North system.

## 4. Research Methods

This section contains a description of the original reduction methods that were used during the research: the true bathymetric data reduction method (TBDRed) and Optimum Dataset (OptD) method.

### 4.1. TBDRed Method

The first of the proposed methods is the true bathymetric data reduction method (TBDRed method), which is described in detail in the article [17]. Based on previous research, the TBDRed method for reducing the bathymetric dataset was carried out through the following steps:

1. The initiatory partition of the individual input data domains into quadrats. In this case, the maximum size of the resultant square is 100 m × 100 m. The initial division is related to the computing power of standard computers, which are used to process bathymetric data;
2. The partition of the region overlaid by bathymetric points. This is closely related to the scale of the final hydrographic product. At this stage, the method indicates the size of the cluster, which is dependent on the function with empirically selected coefficients. The vertical axis shows the size of the cluster presented in square meters and the horizontal axis represents the scale.

f(x) = 2.1x^2^ − 5.2x + 4.49 (1)

3. The desired parameter, obtained by the function (1), is divided by a number of squares (Supplementary Materials Table S1). This depends on the settings used and the size of the input area in the initiatory partition;
4. The extraction of the square root in each case from previously received parameters.
5. Here, the parameter K is taken into account. Parameter K relates to the number of clusters obtained using an Artificial Neural Network with a Kohonen network, also called a Self-Organizing Map. In the case of self-organizing Kohonen network learning, there is no relationship between input indicators and the output of the network. While using this network, the competition between predefined neurons provides the basis for updating values assigned to their weights. In previous studies, the optimal parameters of the self-organized artificial neural network for the reduction method were clearly indicated: an initial neighborhood size of 10, Euclidean distance analysis, a hexagonal topology, 200 iterations during processing, and the Winner Take Most rule;
6. The indication of the optimal value for parameter C, which corresponds to twice the maximum depth in each tested area [17].

The TBDRed method allows bathymetric points to be reduced while maintaining the real value of depth points with the real latitude and longitude. Its application allows the necessary accuracy of surface mapping to be maintained. The bathymetric points after reduction are evenly distributed and significantly reduced. The parameters of the method are only dependent on the scope of input spatial data and the depth of the tested area [17].

### 4.2. OptD Method

The second proposed method is the OptD method. The main aim of the OptD method is to reduce the dataset of measurement observations [18,19]. The degree of reduction is determined by setting the optimization criterion (f) (percent of points or the number of points in the dataset after reduction). Previous applications of the OptD method for processing the MBES dataset in almost real time were proposed in [20]. The OptD method was modified for this purpose. This modification relies on introducing a loop in the OptD method for fragmentary data processing. The OptD method employed for reducing the MBES dataset in this study was carried out through the following stages:

1. Reading the MBES dataset;
2. Setting the optimization criterion (f);
3. Dividing the processing area (in the 2D plane, projection onto a plane from above) into measurement strips. The strips can be horizontal (OXY) or vertical (OYX). The width of the measurement strip is automatically calculated (without the user’s participation) and adjusted in subsequent iterations;
4. Each measurement strip is analyzed separately with the use of the Douglas–Peucker generalization algorithm [21]. The generation of lines created by points in the measurement strips is always performed in the OXD or OYD plane (where D is the depth). Therefore, the changes of depth are detected. The way in which the points are tested in the context of being removed or preserved in the dataset depends on the tolerance range (t) related to the chosen cartographic generalization method;
5. The end of the OptD processing occurs when the generalization algorithm is applied in all measurement strips;
6. The optimum 3D multibeam echosounder dataset is saved in an output file. The saved dataset will meet the optimization criterion determined in stage 2.

It is important to mention that, during processing with the OptD method, only the optimization criterion (f at the second stage) depends on the user. The other parameters, such as the range of tolerance during the Douglas–Peucker algorithm and the width of the measurement strip, are determined in the iteration process. Therefore, these values are changed during iteration until the output dataset meets the optimization criterion.

The main assumption of the OptD method is the reduction of points in a big data set by removing non-characteristic points that may be omitted in the main processing stage, e.g., during the generation of digital models. The OptD method algorithm checks each observation for its suitability for further elaboration. This examination is usually carried out using additional information about the point, such as the depth, height, temperature, and intensity. The method can be used to reduce the sets from LiDAR for the purposes of, e.g., detecting defects in walls [21], reducing the redundancy of data from mobile devices [22]. The method was also used to reduce the real-time reduction of MBES observations [23]. Research has shown that the navigator has full control over the number of observations and the digital models obtained have a good quality. In the case of an isoline map, the obtained results show that isoline maps generated after the OptD method are more readable and isolines present more visible depths.

## 5. Methodology and Research

The research process consisted of four main stages: data acquisition and initial processing; dataset reduction by the TBDRed method and OptD method; results analysis; and the fusion of methods and results.

In the first stage, bathymetric data from two selected test areas were prepared. The data were properly processed, cut, and converted to a text format. As a result, two sets of data were created, which contained the exact position and depth for each of the measurement points.

In the next step, the authors reduced the data with two own reduction methods: the TBDRed method and OptD method. Three different settings were selected for each method. These settings were optimized equally for both methods. The output parameters were the settings of the TBDRed method. According to the method’s operation, three different settings were assumed, in order to obtain three reduced sets of bathymetric data on three different levels. The following settings were produced:

1. Setting no. 1: scale 1:500 for the TBDRed method, which corresponds to the reduction degree at the level of 99.85% for test area no. 1 and 96.26% for test area no. 2 in the OptD method;
2. Setting no. 2: scale 1:1000 for the TBDRed method, which corresponds to the reduction degree at the level of 99.95% for test area no. 1 and 98.79% for test area no. 2 in the OptD method;
3. Setting no. 3: scale 1:2000 for the TBDRed method, which corresponds to the reduction degree at the level of 99.98% for test area no. 1 and 99.70% for test area no. 2 in the OptD method.

After the reduction, the resulting sets were analyzed and on this basis, the parameters for the OptD method were determined. In this case, the main criterion was the number of reduced points. It was assumed that the resulting sets of the second method should have the same number of points as the sets obtained with the first method. As a result, 12 different sets of reduced data were obtained. These 12 combinations are presented in Table 1.

**Figure 3:**
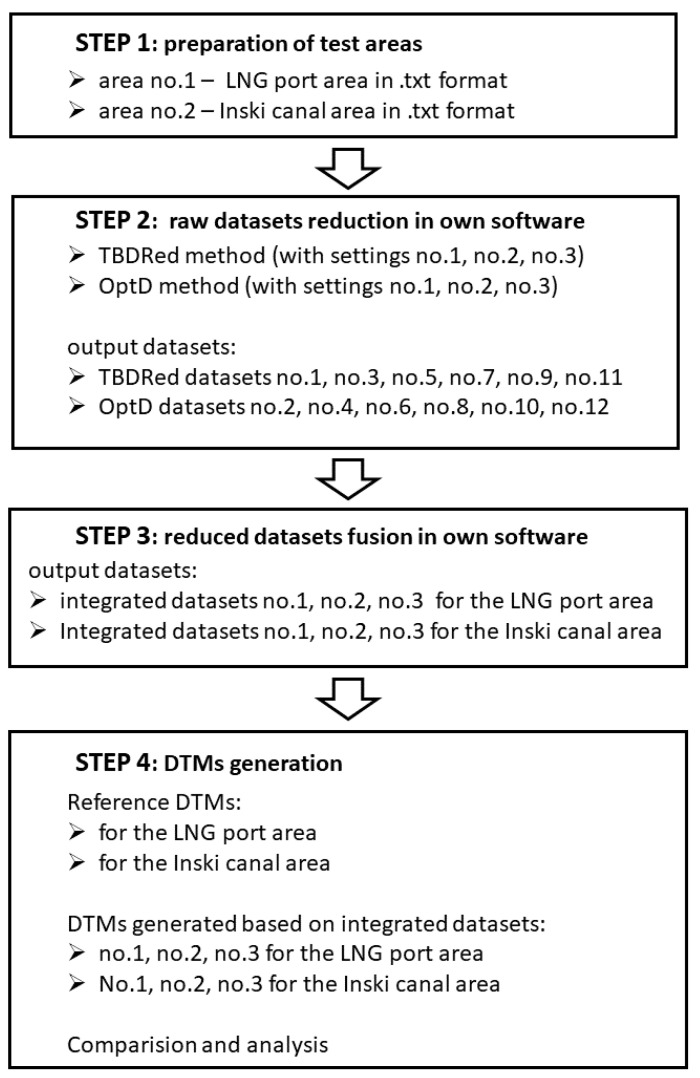
The research methodology.

In the next step, the authors compared the resulting sets. The comparison was based on statistical and visual analysis. The last step carried out during the research was the fusion of bathymetric data obtained by the two proposed methods. The result was six data sets after reduction: three sets for the LNG port area and three sets for the Inski canal area. The main research methodology is schematically presented in Figure 3.

The reduction of bathymetric data was done with the use of copyright programs. Specialized hydrographic software GeoSwatch+ was used to process the bathymetric data. Additionally, ArcGIS software and Surfer software were used to analyze and visualize spatial data.

## 6. Results

The two test areas were processed by the TBDRed and OptD methods. The datasets were reduced using three settings, which are presented in Table 1. The bathymetric data from this area has been reduced and visualized. In addition, the authors performed a statistical analysis of reduced datasets.

### 6.1. Processing by the TBDRed Method

**Figure 4:**
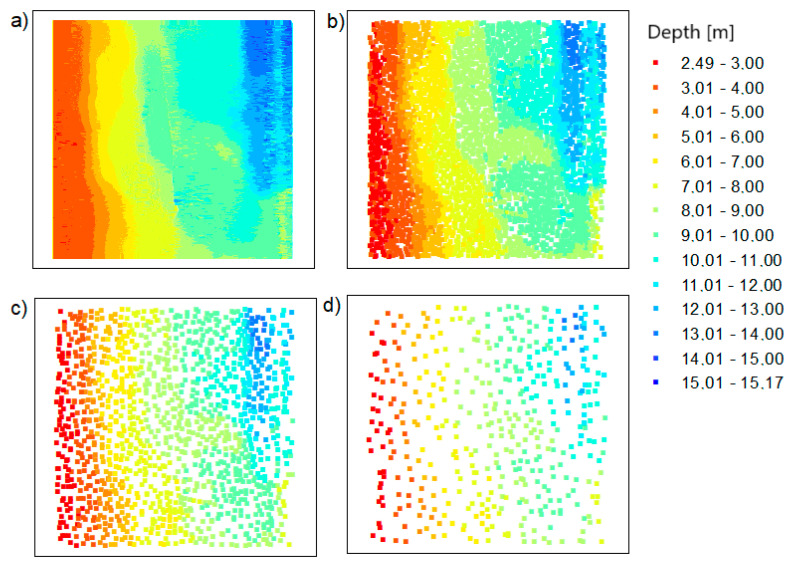
The points based on the LNG port area: (**a**) raw data; (**b**) dataset reduced with the true bathymetric data reduction method (TBDRed) method for setting no. 1; (**c**) dataset reduced with the TBDRed method for setting no. 2; (**d**) dataset reduced with the TBDRed method for setting no. 3.

First of all, the data for test area no. 1 were reduced. As mentioned earlier, the data were reduced for three different method parameter settings. Figure 4 shows a visualization of the spatial distribution of points after reduction.

All bathymetric points retained their position and actual depth. Reduced spatial points were evenly distributed. This is closely related to the assumptions for applying the method. The shape of the bottom surface was preserved.

In addition to visual evaluation, the authors performed a statistical analysis of reduced datasets. All statistics are presented in Table 2.

The number of points, minimum depth, maximum depth, mean depth, standard deviation, and data reduction level expressed as a percentage (this is the number of reduced points) were determined. Additionally, the frequency distribution is presented as a graph. By analyzing the above table, we can see that the data sets were significantly reduced. In the first case, the set was reduced by 99.85%; in the second case, by 99.95; and in the third, by 99.98%. In all research cases, the minimum depth is equal to the minimum depth of the input data, which means that the method focuses on the safety of navigation. The maximum depth values range from 13.32 to 13.55 m. The mean depth in each case is greater than the average entry depth. The last analyzed parameter is the standard deviation, which is between 2.64 and 2.71.

**Figure 5:**
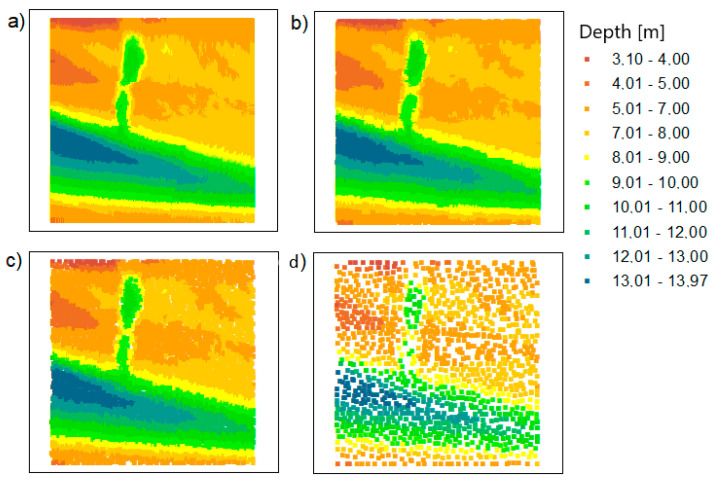
The points based on the Inski canal area: (**a**) raw data; (**b**) dataset reduced with the TBDRed method for setting no. 1; (**c**) dataset reduced with the TBDRed method for setting no. 2; (**d**) dataset reduced with the TBDRed method for setting no. 3.

During data reduction using the TBDRed method for test area no. 2, the distribution of bathymetric points shown in Figure 5 was obtained. In accordance with the reduction method used, the output points are evenly distributed over the test surface.

In the case of the second test area, the data sets were also significantly reduced. It should be remembered that, in this case, the test area is larger—it is 40,000 m^2^. However, the input data set is smaller than in the case of the first examined area. In the first case, the datasets were reduced by 96.24%; in the second case, by 98.81%; and in the third, by 99.68%. The statistical analysis is presented in Table 3.

In all cases, the minimum depth is equal to the minimum input depth. The average depth in each case is less than the average input depth. The value of standard deviation increases slightly as the level of reduction increases, but is, in any case, smaller than the input value. By summarizing the operation of the reduction method, we can clearly state that it meets all of its assumptions.

### 6.2. Processing by the OptD Method

**Figure 6:**
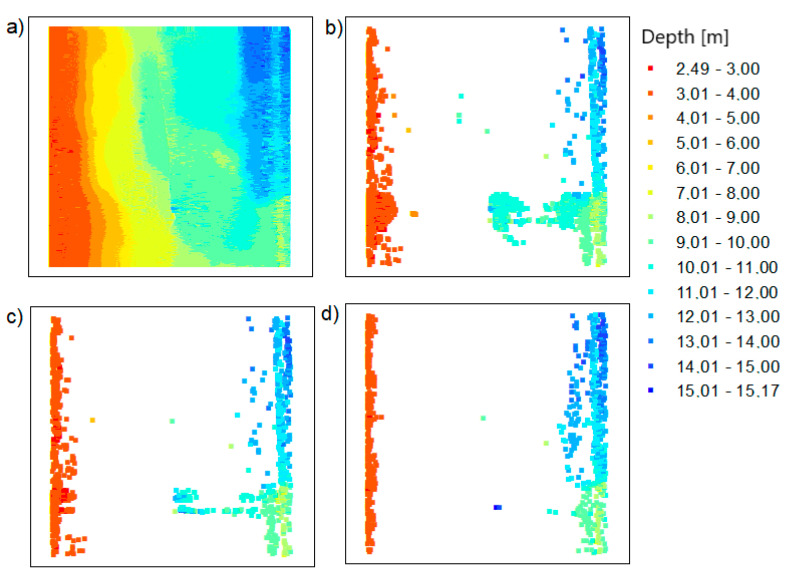
The points based on the LNG port area: (**a**) raw data; (**b**) dataset reduced with the Optimum Dataset (OptD) method for setting no. 1; (**c**) dataset reduced with the OptD method for setting no. 2; (**d**) dataset reduced with the OptD method for setting no. 3.

In the next step of the methodology, the LNG port area and Inski canal area measurement datasets were reduced using the OptD method. In order to obtain similar results to the TBDRed method, the optimization criterion in the OptD method was adopted as the number of points in the dataset (*f* = number of points (No.P)). In this way, the examination of points in the subsequent measurement strips (L) was started. The tests were carried out in the OXH plane, which allowed the extreme depth values to be maintained. Figure 6 presents the results after using the OptD method for the LNG port area.

In Figure 6, the arrangement of points in the datasets after reduction is different than in Figure 4. That means that the two methods give different results, which can be joined. After applying the OptD method, the points only remained in places where the bottom shape changed, leaving places with extreme depths.

The characteristics of the obtained dataset are presented in Table 4.

The analysis of the parameters after reduction presented in Table 4 shows that the obtained numbers of points were similar to those obtained for the TBDRed method. Maximum depth (MaxD) and minimum depth (MinD) values were maintained for all scenarios. Only for the dataset with setting no. 3 did the MinD value change. However, in this case, only about 0.02% of the points in the set remained, and the OptD method retained points at the boundaries of the tested object (depending on the adopted plane). This means that the OptD reduction method is limited in terms of the number of points, and each reduction has its own limit. In this case, it seems reasonable to supplement the dataset with points that come from a different reduction method. The dataset will still contain a small number of points, but these will be points of great importance when generating the digital model.

**Figure 7:**
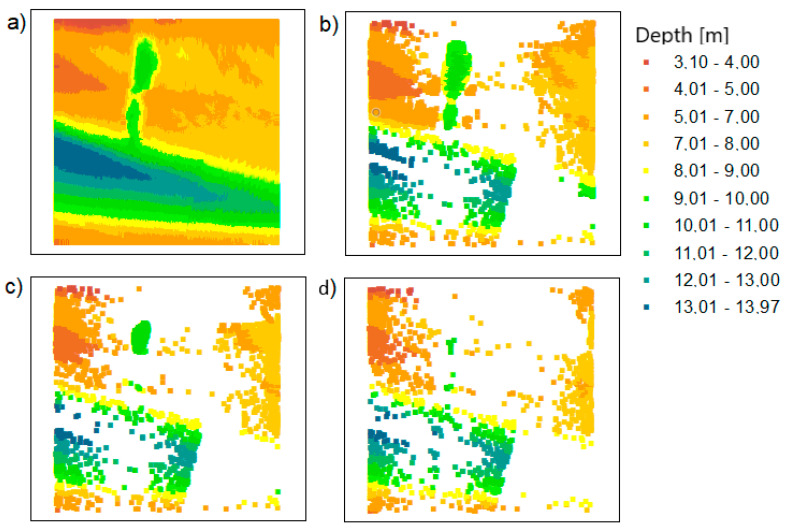
The points based on the Inski canal area: (**a**) raw data; (**b**) dataset reduced with the OptD method for setting no. 1; (**c**) dataset reduced with the OptD method for setting no. 2; (**d**) dataset reduced with the OptD method for setting no. 3.

Similar reductions were made for test area 2. The results of the OptD method are presented in Figure 7. It shows that the characteristic points of the bottom have been preserved. The places where the depth values are large are visible (the colors in the legend correspond to the values 12.02–13.97 m).

In the case of the OptD method, the points are not evenly distributed. There are more points in areas with significant changes in depth.

The characteristics of the obtained dataset are presented in Table 5. The statistics of the obtained datasets after reduction by the OptD method show that the MinD value was maintained in all scenarios. However, the MaxD value changed (similar to TBDRed processing). This is really due to the high degree of reduction (data reduction level (RLev) = 96.26%, 98.79%, and 99.70%).

The datasets after reduction were significantly reduced. It is important, however, that despite the high degree of reduction, it was not accidental. The combination of the results obtained from the two methods is again justified here. With such a high degree of reduction, the user wants to be sure that the obtained dataset can be used to build a digital model.

### 6.3. Combining Outputs

**Figure 8:**
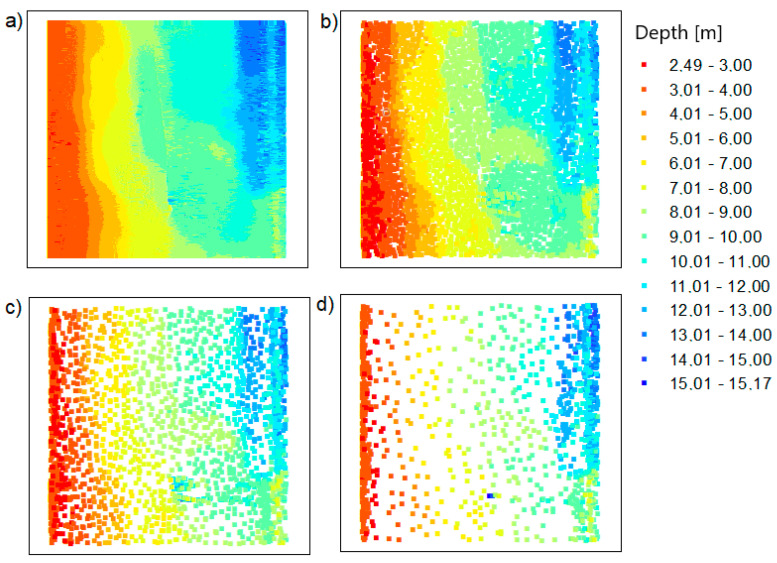
The datasets for the LNG port area: (**a**) raw dataset; (**b**) integrated dataset no. 1; (**c**) integrated dataset no. 2; (**d**) integrated dataset no. 3.

**Figure 9:**
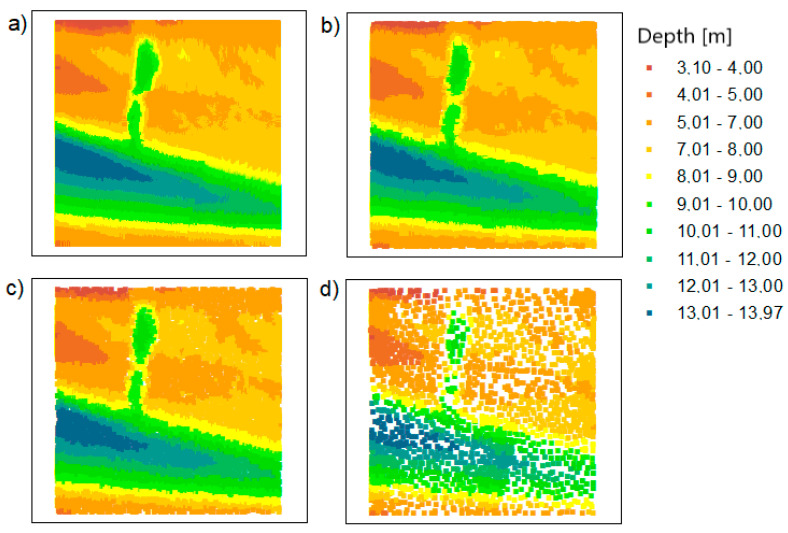
The datasets for the Inski canal area: (**a**) raw dataset; (**b**) integrated dataset for setting no. 1; (**c**) integrated dataset for setting no. 2; (**d**) integrated dataset for settings no. 3.

The main aim of the research was to fuse the reduced data using two original methods. The authors integrated the data in their proprietary software to obtain the final bathymetric datasets containing the actual values of X, Y, and D coordinates:

1. TBDRed dataset no. 1 + OptD dataset no. 2 = integrated dataset no. 1 for the LNG port area (presented in Figure 8b);
2. TBDRed dataset no. 3 + OptD dataset no. 4 = integrated dataset no. 2 for the LNG port area (presented in Figure 8c);
3. TBDRed dataset no. 5 + OptD dataset no. 36 = integrated dataset no. 3 for the LNG port area (presented in Figure 8d);
4. TBDRed dataset no. 7 + OptD dataset no. 8 = integrated dataset no. 1 for the Inski canal area (presented in Figure 9b);
5. TBDRed dataset no. 9 + OptD dataset no. 10 = integrated dataset no. 2 for the Inski canal area (presented in Figure 9c);
6. TBDRed dataset no. 11 + OptD dataset no. 12 = integrated dataset no. 3 for the Inski canal area (presented in Figure 9d).

Integrated datasets do not contain points—duplicates. The TBDRed and OptD methods leave different points in the datasets, but do not exclude the possibility that some of them will be the same. This procedure is used to keep the file size as small as possible.

The resulting reduced bathymetric datasets for both test areas are presented in Figure 8 and Figure 9.

The collection numbers are presented in Table 6.

The results presented in Table 6 confirm the effectiveness of dataset fusion and the reliability of the obtained results. The extreme depth values were maintained for all datasets. The combination of files also improved the operation of individual methods, e.g., for the LNG port area, after TBDRed processing (Table 2), MaxD was not preserved in relation to the original file. However, after combining the integrated datasets ultimately numbers of points (No.U) = 1, 2, and 3, this depth was maintained.

The MinD value is the same for individual datasets, while in the case of MaxD for the LNG port area, it is the same, and for the Inski canal area, it is different and takes the maximum value from the method in which it was left in the dataset.

## 7. DTM Generation and Discussion

**Figure 10:**
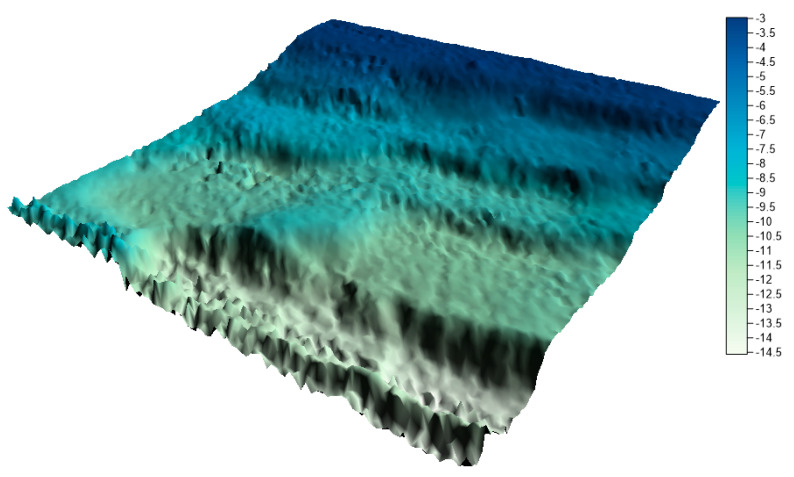
Reference surface for the LNG port area.

In the next step, on the basis of all collections, numerical bottom models were generated and compared with the reference surface. In his study, Malejka [24,25] showed that, only taking into account the accuracy of the numerical bottom model, the best interpolation method for data is Kriging. The authors decided to create a reference surface using this method and to apply the mesh size of 1 m. This is a standard resolution used by hydrographic offices all over the world when creating numerical bottom models. The visualization of the reference surface for the LNG port area is shown in Figure 10.

**Figure 11:**
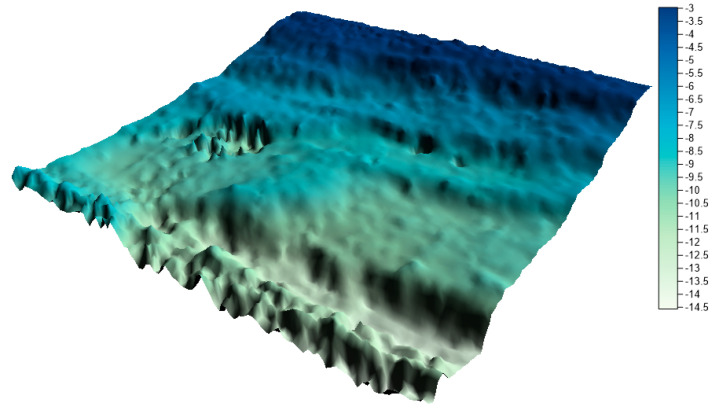
Surface obtained by integrated dataset no. 1 for the LNG port area.

**Figure 12:**
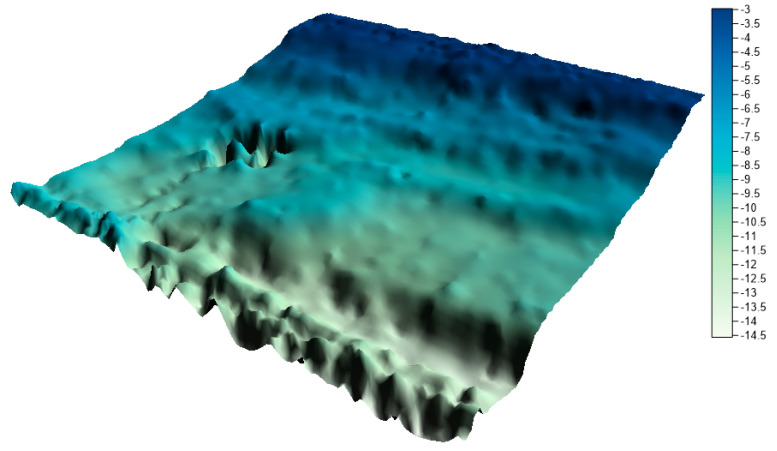
Surface obtained by integrated dataset no. 2 for the LNG port area.

**Figure 13:**
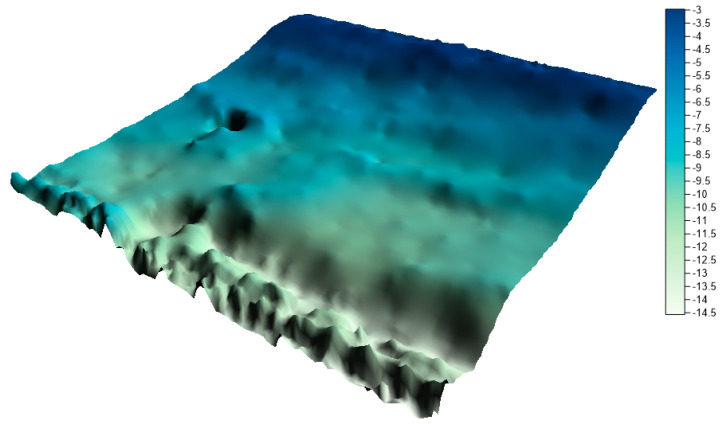
Surface obtained by integrated dataset no. 3 for the LNG port area.

The surfaces created from the integrated data sets for the LNG port area are shown in Figure 11, Figure 12 and Figure 13 below.

By visually analyzing the obtained surfaces, it can be concluded that the character of the bottom has been preserved for all models generated from the integrated datasets. The greatest depression is still visible. When analyzing the above models, it should be remembered that the main problem to be solved during reduction methods used was a very high degree of reduction. The models were generated on the basis of approximately 0.3% (integrated dataset no. 1 for the LNG port area), 0.1% (integrated dataset no. 2 for the LNG port area), and 0.04% (integrated dataset no. 1 for the LNG port area) points in the dataset compared to the raw dataset.

The characteristics of the digital models generated are presented in Table 7. We calculated the differences in the depth values among the input data before the reduction and the values obtained for the modeled surfaces. Then, we calculated their absolute values and statistical parameters: the minimum error; maximum error; mean error; and standard deviation.

The minimum error is zero in each case. The maximum error is in the range from 4.98 to 5.71 m. This is closely related to the large number of reduced points. The methods focused on the shallowest depth values of the bottom and those points that were deep and insignificant for navigation were reduced. The mean error value for each setting is between 0.39 and 0.53 m. Taking into account the characteristics of the sea bottom shape and the level of reduction, the errors are not large, whereas errors related to the reflection of the laser beam from low vegetation, pollution, or sea currents cause continuous changes in the shape of the bottom. For measurements such as the object terrain, bottom, and depth errors of up presented values can be treated as not large. The highest standard deviation was obtained for the integrated dataset for setting no. 3, but it can be considered relatively small in terms of the considered area, which is the bottom. However, for the integrated dataset for setting no. 1, the standard deviation is only 0.19 m.

**Figure 14:**
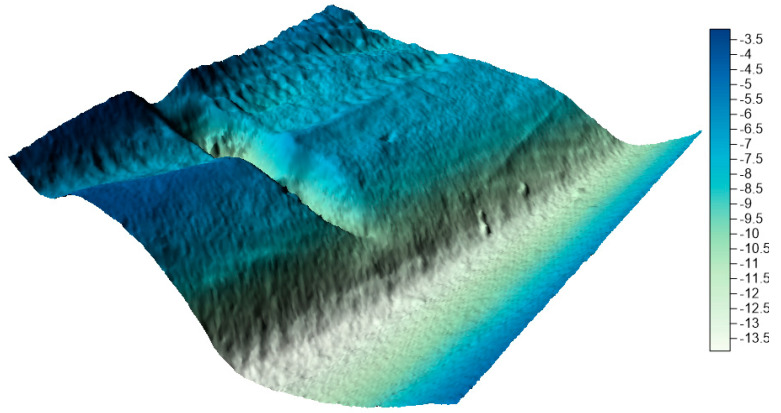
Reference surface for the Inski canal area.

**Figure 15:**
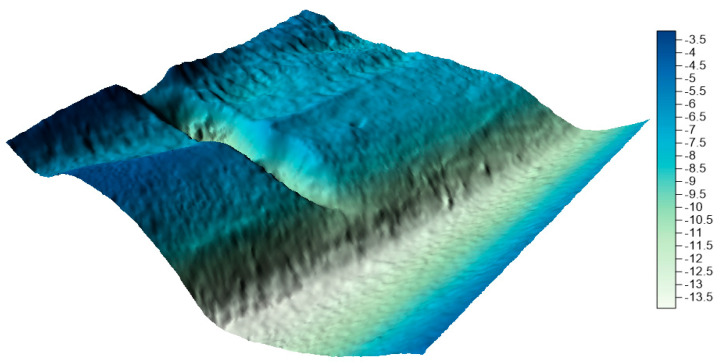
Surface obtained by integrated dataset no. 1 for the Inski canal area.

**Figure 16:**
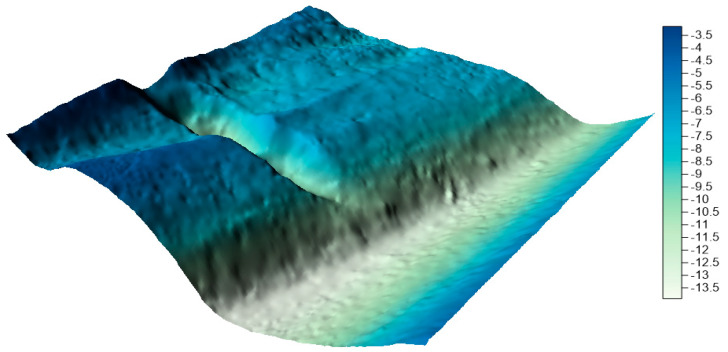
Surface obtained by integrated dataset no. 2 for the Inski canal area.

**Figure 17:**
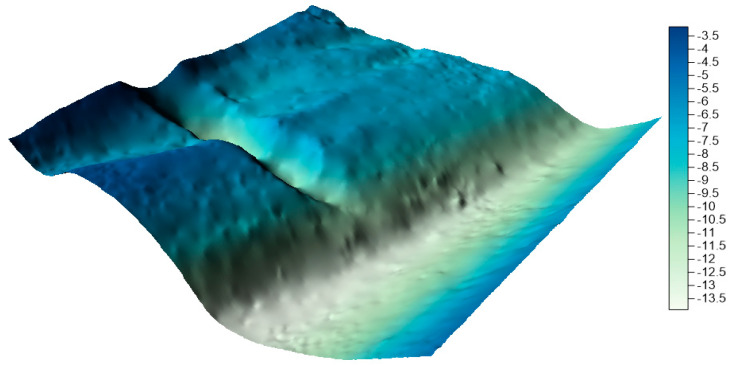
Surface obtained by integrated dataset no. 3 for the Inski canal area.

The test area 2—Inski canal—was also analyzed. The reference numerical model generated is presented in Figure 14, while the models generated on the basis of the integrated datasets correspond to Figure 15, Figure 16 and Figure 17.

On the basis of the visual assessment of the obtained numerical models, it can be concluded that they reflect the model area very well, despite the fact that they were generated on the basis of the datasets integrated after reduction, in which there were 7.5% (integrated dataset no. 1 for the Inski canal area), 2.4% (integrated dataset no. 2 for the Inski canal area), and 0.62% (integrated dataset no. 1 for the Inski canal area) points in the dataset compared to the raw dataset.

The results of the comparison of individual surfaces with the reference surface for the Inski canal area with statistical parameters (minimum error, maximum error, mean error, and standard deviation) are included in Table 8.

For the second test area, the errors are smaller. This is related to the size of the input dataset. In this case, the minimum error was also zero. The maximum errors are in the range of 0.65 to 1.08 m. The mean errors are not larger than 9 cm. The standard deviation in the case of the area 2 test ranges from 0.05 to 0.08 m, and is a value of centimeters. Therefore, it is assumed that, for these surfaces, we can observe a very good fit to the reference model.

## 8. Conclusions

This paper presents two methods for reducing large datasets: TBDRed and OptD. These were used to reduce sets of bathymetric observations. As mentioned, the existing software only offers the possibility of reducing the size of the input files by accidental/random reduction. The proposed solution makes it possible to deliberately reduce the dataset of observations and leave only those points that affect the shape and quality of the digital model.

Due to the fact that big datasets are difficult, and sometimes even impossible, to use rationally, after reduction, it is possible to work with small data sets. Many computer programs cannot cope with large amounts of data. Despite the continuous improvement of computer hardware, many users do not have access to the latest computers with high computing power due the high cost of purchasing them. Therefore, it seems rational to reduce the data. A small number of points in the collections allows for more efficient processing and the specification of the characteristic features of the object. Redundant information is omitted and only meaningful information is left in the resulting dataset.

As discussed in Section 3, the methods work differently. The TBDRed method allows for a more even distribution of points, while the OptD method allows for a different degree of reduction in different areas of the tested object.

Users often cannot control the reduction result. Using the reduction methods existing in the programs, e.g., high reduction, medium reduction, and low reduction, the user does not know how many points will be maintained in the dataset after reduction. The proposed methods make it possible to determine exactly how many points the user wants to receive after the reduction. The fusion of two proven methods, operating on different principles, but with the same aim (to achieve the minimum number of points in the dataset with the assumed optimization criterion), is an excellent solution. Combining the results of the two methods allows for the preservation of characteristic points in the studied area, and then for the generation of a digital bottom model (Figure 11, Figure 12 and Figure 13 and Figure 15, Figure 16 and Figure 17) with a quality that does not differ much from the reference model (Figure 10, Figure 14).

The main advantages of the proposed fusion include the following:

(a) The even distribution of points;
(b) Maintaining extreme depths;
(c) The total control over the number of observations in the input dataset;
(d) Improving the efficiency and speed of generating digital models;
(e) The convexity, improving the visibility of the characteristic features of the area (more visible concavities and convexities);
(f) Keeping only those points that are important from the point of view of further purposes in the integrated dataset;
(g) Reducing the memory occupied by files.

Thanks to the above advantages, the obtained integrated collections meet the criteria included in the latest international regulations.

The OptD and TBDRed methods control and complement each other.

The proposed fusion of reduction methods can be the basis for methods related to the creation of large numerical seabed models, as well as in the processing, analysis, and visualization of big spatial datasets that consist of real measurements obtained by modern hydroacoustic sensors. In future steps, the authors plan to use the new proposed methodology of processing MBES datasets for the extraction of objects located at the bottom of waters. The proposed fusion of the TBDRed and OptD methods can improve the process of extraction points belonging to objects and speed up object detection.

## Figures and Tables

**Table 1:** Settings used and possible resulting sets.

Test Area	TBDRed	No. Dataset	OptD	No. Dataset
LNG port area	setting no. 1	1	setting no. 1	2
	setting no. 2	3	setting no. 2	4
	setting no. 3	5	setting no. 3	6
Inski canal area	setting no. 1	7	setting no. 1	8
	setting no. 2	9	setting no. 2	10
	setting no. 3	11	setting no. 3	12

**Table 2:** Statistics for points reduced with the TBDRed method based on the LNG port area.

	Raw Dataset	Reduced Dataset for Setting No. 1	Reduced Dataset for Setting No. 2	Reduced Dataset for Setting No. 3
No.P	2m670,449	4096	1320	426
FD	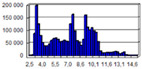	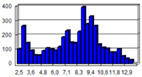	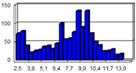	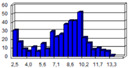
MinD	2.49	2.49	2.49	2.49
MaxD	15.17	13.55	13.35	13.32
MeanD	7.30	7.91	7.79	7.99
SD	2.64	2.71	2.70	2.64
RLev	---	99.85	99.95	99.98

No.P—number of points, FD—frequency distribution, MinD—minimum depth (m), MaxD—maximum depth (m), MeanD—mean depth (m), SD—standard deviation, RLev—data reduction level (%).

**Table 3:** Statistics for points reduced with the TBDRed method based on the Inski canal area.

	Raw Dataset	Reduced Dataset for Setting No. 1	Reduced Dataset for Setting No. 2	Reduced Dataset for Setting No. 3
No.P	435,804	16,379	5188	1376
FD	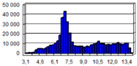	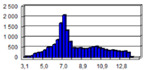	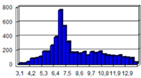	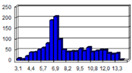
MinD	3.10	3.10	3.10	3.10
MaxD	13.97	13.84	13.83	13.73
MeanD	8.77	8.31	8.24	8.26
SD	2.55	2.32	2.34	2.43
RLev	---	96.24	98.81	99.68

No.P—number of points, FD—frequency distribution, MinD— minimum depth (m), MaxD—maximum depth (m), MeanD—mean depth (m), SD—standard deviation, RLev—data reduction level (%).

**Table 4:** Statistics for points reduced with the OptD method based on the LNG port area.

	Raw Dataset	Reduced Dataset for Setting No. 1	Reduced Dataset for Setting No. 2	Reduced Dataset for Setting No. 3
No.P	2,670,449	3976	1312	591
FD	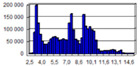	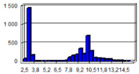	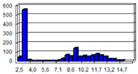	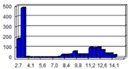
MinD	2.49	2.49	2.49	2.73
MaxD	15.17	15.17	15.17	15.17
MeanD	7.30	7.23	7.16	8.97
SD	2.64	3.53	3.91	4.28
RLev	---	99.85	99.95	99.98

No.P—number of points, FD—frequency distribution, MinD—minimum depth (m), MaxD—maximum depth (m), MeanD—mean depth (m), SD—standard deviation, RLev—data reduction level (%).

**Table 5:** Statistics for points reduced with the OptD method based on the Inski canal area.

	Raw Dataset	Reduced Dataset for Setting No. 1	Reduced Dataset for Setting No. 2	Reduced Dataset for Setting No. 3
No.P	435,804	16,282	5272	1302
FD	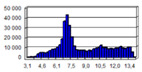	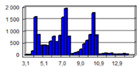	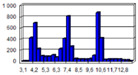	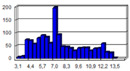
MinD	3.1	3.1	3.1	3.1
MaxD	13.97	13.95	13.67	13.85
MeanD	8.77	7.55	7.57	7.79
SD	2.55	2.26	2.52	2.52
RLev	---	96.26	98.79	99.70

No.P—number of points, FD—frequency distribution, MinD—minimum depth (m), MaxD—maximum depth (m), MeanD—mean depth (m), SD—standard deviation, RLev—data reduction level (%).

**Table 6:** Statistics for integrated datasets.

Test Area	Number of Integrated Dataset	No.P	No.D	No.U	MaxD	MinD
LNG port area	1	8072	886	7186	15.17	2.49
	2	2632	584	2048	15.17	2.49
	3	1017	158	859	15.17	2.49
Inski canal area	1	3266	7020	25,641	13.95	3.10
	2	10,460	1205	9255	13.84	3.10
	3	2678	250	2428	13.85	3.10

No.P—number of points, No.D—number of duplicates, No.U—ultimately numbers of points, MinD—minimum depth (m), MaxD—maximum depth (m).

**Table 7:** Error values: analysis of the resulting surfaces compared to the reference surface for the LNG port area.

	Integrated Dataset No. 1	Integrated Dataset No. 2	Integrated Dataset No. 3
Minimum Error (m)	0.00	0.00	0.00
Maximum Error (m)	5.71	4.98	5.55
Mean Error (m)	0.39	0.45	0.53
Standard deviation (m)	0.19	0.25	0.31

**Table 8:** Error values: analysis of the resulting surfaces compared to the reference surface for the Inski canal area.

	Integrated Dataset No. 1	Integrated Dataset No. 2	Integrated No. 3
Minimum Error (m)	0.00	0.00	0.00
Maximum Error (m)	0.65	0.70	1.08
Mean Error (m)	0.06	0.07	0.09
Standard deviation (m)	0.05	0.06	0.08

## Supplementary Materials

The following are available online at https://www.mdpi.com/1424-8220/20/21/6207/s1, Table S1: The number of subsets depending on the data range and the research parameters.
